# Camera traps reveal that terrestrial predators are pervasive at riverscape cold‐water thermal refuges

**DOI:** 10.1002/ece3.10316

**Published:** 2023-07-17

**Authors:** Christopher J. Sullivan, Chadwick D. Rittenhouse, Jason C. Vokoun

**Affiliations:** ^1^ Department of Natural Resources and the Environment, Wildlife and Fisheries Conservation Center University of Connecticut Storrs Connecticut USA

**Keywords:** camera trap, predator, riverscape, salmonid, thermal refuge, visitation rates

## Abstract

Perceived predation risks by terrestrial predators are major ecological forces in aquatic systems, particularly for aggregating fish. Riverscape thermal refuges are discrete, localized cold‐water patches where fish temporarily aggregate to buffer against heat events. Predation pressures by terrestrial predators at thermal refuges may decrease the thermoregulatory benefits of refuge use, but quantifying such effects can be challenging and controversial when sampling can impose additional stress on fish. We passively monitored terrestrial predator visitation patterns and predation at four thermal refuges in the Housatonic River, Connecticut, USA, between May 18th and September 29th, 2022, with camera traps, a common wildlife monitoring method. Specifically, we (1) assessed diel visitation patterns by different categories of terrestrial predators at thermal refuges and determined if patterns varied among predator categories or with prevailing environmental conditions, and (2) estimated the probability of predation by hour of the day combined across all predator categories, quantifying general predation pressures at refuges. We detected at least one terrestrial predator at a thermal refuge each day, and mean hourly visitation rates (count/h) were highly variable across predator categories and sampling dates. The most supported generalized additive mixed model indicated that terrestrial predator visitation rates (count/h/day) varied with mean daily river discharge and water temperature differential, and relationships differed across categories of terrestrial predators. We observed 22 separate predation attempts on thermoregulating salmonids and predicted that the probability of predation by any terrestrial predator increased from 0.002 to 0.017 throughout a 24 h day (*p* = .004). Camera traps provided novel evidence that terrestrial predators are pervasive at riverine thermal refuges, which is relevant for refuge conservation and management globally. We recommend the implementation of a coordinated monitoring network across riverine thermal refuges using camera traps, further enriching our ecological understanding of cumulative predator effects in refuges across complex riverscapes.

## Graphical abstract

Perceived predation risks by terrestrial predators at thermal refuges may decrease the thermoregulatory benefits of refuge use, but quantifying such effects can be challenging and controversial when sampling can impose additional stress on fish. We passively monitored visitation patterns by different types of terrestrial predators at four thermal refuges in the Housatonic River, Connecticut, USA with camera traps, a common wildlife monitoring method. Camera trap surveys found that terrestrial predators were pervasive at riverscape thermal refuges but predation attempts were infrequent, and we recommend the implementation of a coordinated monitoring network across refuges using camera traps for cold‐water fish conservation globally.

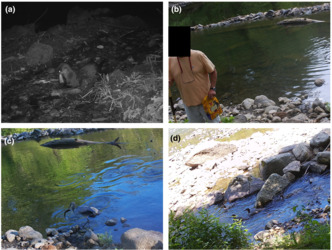

## 1 INTRODUCTION

Perceived predation risks by terrestrial predators are major ecological forces rivaling that of direct predation (i.e., mortality) in aquatic systems (e.g., Preisser et al., 2005). Terrestrial predators can consume large quantities of sub‐adult and adult fish (Budy et al., 2022; Teuscher et al., 2015; Vehanen et al., 2021), influencing fish population size and demographics (Bacheler et al., 2011; Steinmetz et al., 2003), and community structure or food web dynamics (Gagnon et al., 2015; Geodde & Coble, 1981). Individual fish can also respond to perceived predation risks and predation by shifting their behavioral patterns, where individuals can temporarily leave areas and avoid times of the day when terrestrial predators are active (Rahel & Stein, 1988), or shift their habitat use patterns to avoid high‐activity locations (e.g., Doherty et al., 2021). Although these effects manifest in obvious ways through targeted fishery (e.g., trends in abundance) or terrestrial predator (e.g., diets) data collection (e.g., Budy et al., 2022), more subtle are the inconspicuous or unobservable behaviors of predators in aquatic systems, including how their activity and foraging rates vary at short time scales. With the widespread adoption of wildlife monitoring tools such as passive camera traps (Burton et al., 2022; O'Connor et al., 2017), researchers can easily continuously monitor terrestrial predators at a fine resolution and better fill crucial knowledge gaps regarding predator behavioral and foraging dynamics within aquatic systems.

Fish aggregations are gatherings of large numbers of individuals that form for a perceived benefit, including, but not limited to, desired environmental conditions (e.g., Wilbur et al., 2020), reproduction (Budy et al., 2022; Erisman et al., 2017), improved foraging efficiency (e.g., Baker & Foster, 1994), and predation avoidance (Lett et al., 2014), and can be persistent or transient in time. Previous research suggests that perceived predation risks by terrestrial predators on aggregating fish are highly variable but can be extensive in situations where populations are vulnerable, regardless of predator density. Avian predators, for example, are well adapted for hunting fish in littoral or shallow areas in aquatic systems, feeding on and herding fish in locations up to 1 m in water depth (e.g., Sjöberg, 1987), and tend to selectively forage in shallow areas where fish aggregate (e.g., migrating fish; Budy et al., 2022; Levi et al., 2015). Similarly, anglers, another type of terrestrial predator, may preferentially target fish aggregations to maximize catch rates (e.g., Erisman et al., 2011). Not all terrestrial predator impacts on aggregating fish can be direct. For example, aggregating fish may vary in location, schooling orientation, or become skittish in response to the perceived risks by terrestrial predator presence and activity, irrespective of predation rates (Herbert‐Read et al., 2017; Kortan & Adámek, 2011). Such findings underscore the importance of understanding the extent to which aggregating fish may be exposed to perceived predation risks (and mortality) by terrestrial predators, particularly during vulnerable periods or in areas critical for populations.

Thermal refuges are discrete, localized groundwater discharge areas throughout a riverscape where fish temporarily aggregate for metabolic relief (and survival) during extreme heat events (Kurylyk et al., 2015; Sullivan et al., 2021). Areas of groundwater upwellings or interstitial flow, side channels, and lateral springs or seepages systems (Dugdale et al., 2013; Ebersole et al., 2003), and larger confluence zones of groundwater‐dominated tributaries (Baird & Krueger, 2003; Mejia et al., 2020) can serve as an effective thermal refuge. Thermal refuges must be co‐located with the distribution and timing of thermally stressed fish to provide adequate habitat for potentially long‐term occupancy (days to weeks; Baird & Krueger, 2003). Cold‐water‐dependent trout and salmon (hereafter “salmonids”) commonly aggregate in thermal refuges across temperate latitudes for summer survival (e.g., Brewitt et al., 2017; Dugdale et al., 2016). Salmonids aggregate in thermal refuges that are closer in character to their regular habitats throughout the larger riverscape (Petty et al., 2012; Wilbur et al., 2020) but in much higher densities comparatively during a heat event (Ebersole et al., 2001). For example, Morgan and O'Sullivan (2023) found that >1000 salmonids (adult brook trout *Salvelinus fontinalis* and age 0–2 Atlantic salmon *Salmo salar*) aggregated in a single thermal refuge (3–4 m width, 120–130 m length) in the Little Southwest Miramichi River, New Brunswick, Canada as main stem water temperatures approached 31.5°C. Salmonids only aggregate in a minority of available refuges, though, as habitat quality, temperature differential, or connectivity can vary (e.g., Barret & Armstrong, 2022; Wilbur et al., 2020). In some cases, salmonids cannot actualize the lower metabolic costs when exploiting thermal refuges due to perceived risks by terrestrial predators (e.g., Keefer et al.,^2009^; Snyder et al., 2022), but observations are limited. For example, salmonids avoided thermal refuges during the hot late afternoon in two Montana streams as avian predators frequented refuges then (Ritter et al., 2020). As salmonid use of thermal refuges can influence their adaptive capacity to climatic change (e.g., Beever et al., 2017; Lynch et al., 2014), it is critical to quantify terrestrial predator activities, both direct and indirect, more rigorously within thermal refuges at fine time scales.

Addressing terrestrial predator activities and their effects on aggregating salmonids is, however, challenging and can be controversial. Many avian or mammalian terrestrial predators are protected (e.g., The Migratory Bird Treaty Act of 1918) or managed under the direction of state agencies (e.g., hunting/trapping seasons), and surveys of predators can be stressful and involve the handling of many individuals (e.g., collecting predator diets; e.g., Budy et al., 2022). State agencies can also prohibit angler, another category of terrestrial predator, activities within certain times or locations, but illegal angling can be covert and difficult to detect (e.g., nighttime angling; Cooke et al., 2015). Further, conservation activities such as sampling, tagging, and monitoring salmonids aggregating in thermal refuges can impose additional stress on individual fish and temporarily displace fish (Price & Peterson, 2010) or result in unintentional mortality (Snyder, 2003). Rather than regulatory actions or proxy approaches, researchers can use camera traps to survey terrestrial predator activities (behavioral and foraging dynamics) in thermal refuges at fine temporal scales, but we are unaware of any such approaches. A robust investigation into terrestrial predator behavioral and foraging patterns on aggregating salmonids within thermal refuges using camera traps would provide the first quantification of predator activities around refuges and novel insights into the changing ecology and population trajectories of cold‐water‐dependent fish under climate change.

Here, we explored terrestrial predator behavior and foraging patterns on aggregating salmonids at thermal refuges in the Housatonic River, Connecticut and monitored using camera traps during the summertime. Native populations of cold‐water‐dependent brook trout and introduced brown trout *Salmo trutta* and rainbow trout *Oncorhynchus mykiss* support important socioeconomically recreational fisheries in the Housatonic River, and it is currently one of the state's premier fishing destinations. Ongoing climate change is warming the Housatonic River and creating more frequent and extreme heat events, and trout must use thermal refuges for days to months for summer survival. The Connecticut Department of Energy and Environmental Protection (CT DEEP) statewide strategic trout management plan prioritizes protecting cold‐water areas like thermal refuges for long‐term conservation goals (CT DEEP, 2022).

As such, our objectives were to: (1) assess terrestrial predator behavioral, or visitation, patterns at thermal refuges, focusing on diel patterns and (2) estimate the probability of a predation attempt at a refuge by hour of the day across all categories of predator, quantifying general predation pressures at thermal refuges. We hypothesize that different species of terrestrial predators will readily visit and forage in thermal refuges when brown, brook, and rainbow trout are aggregating, and visitation rates will vary with environmental conditions. We predict higher terrestrial predator visitation rates, regardless of species, when main stem water temperatures are high as salmonids aggregate more densely then (O'Sullivan et al., 2023) and when river discharge is low because thermal refuge water depth, velocity, and clarity should be more optimal for predator foraging or angling efficiency (e.g., Sjöberg, 1987). We also include covariates describing air temperature and wind speeds since both can influence feeding behaviors and activities of terrestrial predators (e.g., Stalmaster & Gessaman, 1984). We defined a terrestrial predator as any mammal or avian species known to depredate adult, cold‐water‐dependent salmonids. We also included anglers and non‐anglers as terrestrial predators because anglers illegally harvest aggregating fish, and reports of people illegally netting fish and swimming in thermal refuges are common along the Housatonic River (M. Humphreys, *personal communication*). In doing so, our classification expands the typical use of “terrestrial predator” to allow for the shared expectation that predation pressures can be both human‐ and animal‐induced for riverscape thermal refuges.

## 2 MATERIALS AND METHODS

### 2.1 Study area

**FIGURE 1:**
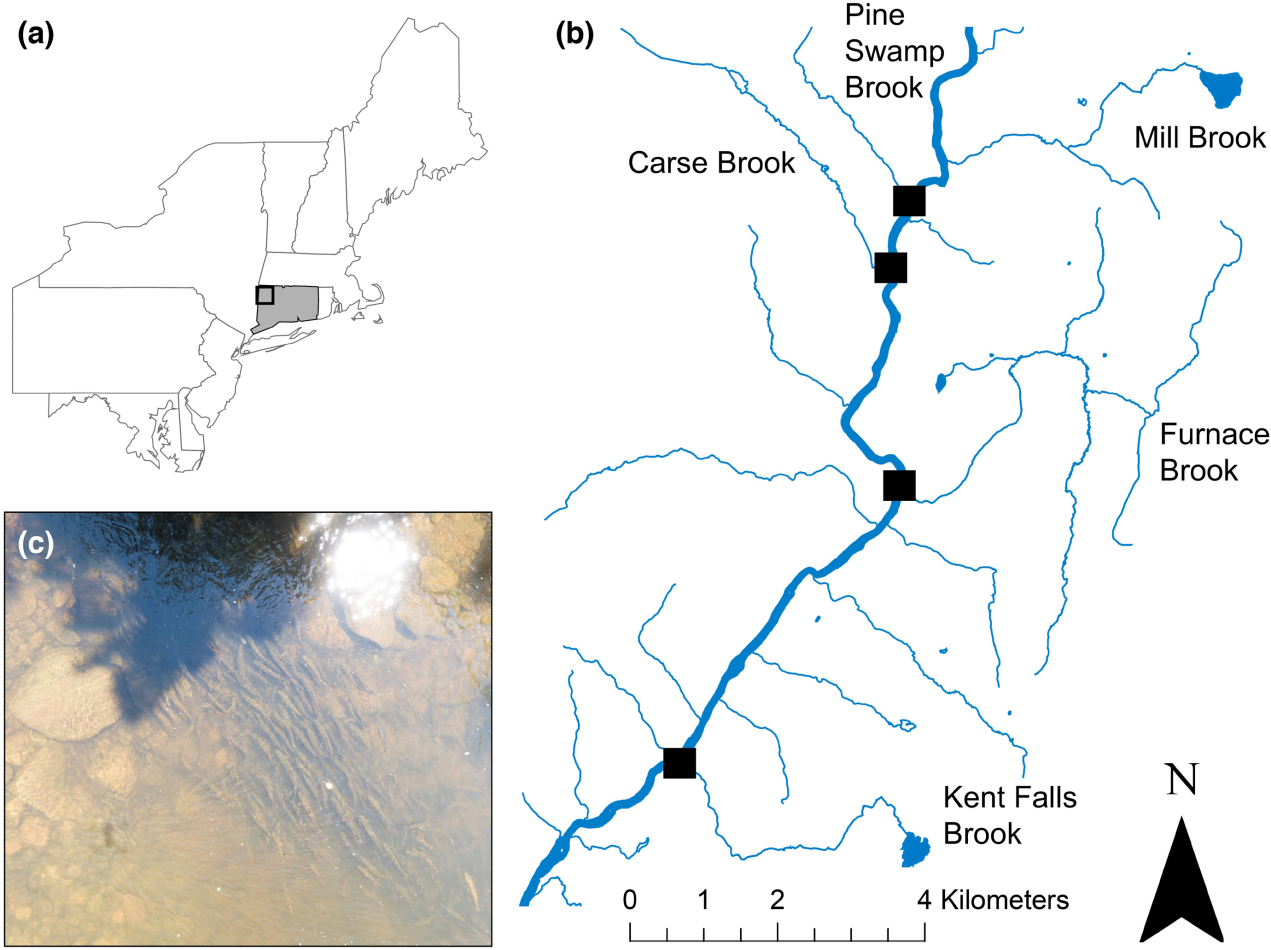
(a) Study area within the Housatonic River, Connecticut, USA. (b) Individual thermal refuges passively monitored using camera traps between May 18th and September 29th, 2022. (c) An aggregation of brown and rainbow trout within the Furnace Brook thermal refuge during our study period.

The Housatonic River originates in western Massachusetts and drains a total area of 5100 km^2^. We focused our sampling efforts within a 15.0 km segment of the Housatonic River between Kent Falls Brook and Mill Brook (Figure 1). This segment of the Housatonic River is characterized by a gentle, sloping topography with several sinuous river sections where incised, semi‐confined channel beds are present. The upstream 9.5 km segment is a designated Trout Management Area (TMA), whereas the remaining 5.5 km downstream segment is not. Within the TMA, CT DEEP only allows catch‐and‐release angling and stocks approximately 18,000 adult brown trout biannually to supplement the fishery. Small private angling clubs stock nearly 5000 adult rainbow trout annually. Several cold‐water tributary confluences provide thermal refuge for brown trout, rainbow trout, and brook trout throughout the 15.0 km study area. We limited sampling to four thermal refuges associated with tributary confluences: Kent Falls Brook, Furnace Brook, Carse Brook, and Pine Swamp Brook (Figure 1). More thermal refuges are present in the study area (e.g., Mill Brook), but we selected thermal refuge sites based on their accessibility for passive monitoring and biologist recommendations. CT DEEP prohibits angling within 100 ft of the four thermal refuges from June 1st to September 15th and conservation officers routinely check for illegal activities at refuges and surrounding river reaches.

### 2.2 Field sampling

We monitored thermal refuges between May 18th and September 29th, 2022, when Housatonic River maximum water temperatures exceeded 20°C daily. Time‐lapse underwater camera data from Furnace Brook and Mill Brook suggest that brown, brook, or rainbow trout occupied thermal refuges from July 1st to September 6th (C.J. Sullivan, *unpublished data*), representing a large portion of our study period. We used wildlife camera traps (Trophy Cam HD Essential) to monitor terrestrial predators continuously at each thermal refuge. We deployed one to three camera traps at each thermal refuge to ensure sufficient coverage: Pine Swamp Brook (*n* = 2), Carse Brook (*n* = 1), Furnace Brook (*n* = 3), and Kent Falls Brook (*n* = 3). We placed each camera trap at approximately 1.5 m height and facing toward the thermal refuge. We avoided any vegetation or object that would severely obstruct the camera image or cause false‐trigger events. We set passive‐infrared sensors to medium sensitivity and to capture three images per trigger event at a 60 s interval. We equipped each camera trap with a 32 GB SD card and housed each camera in a commercially available locking case. We visited cameras monthly to exchange memory cards and change out batteries. We also monitored water temperatures within each thermal refuge and the main stem Housatonic River using HOBO UA‐002‐64 loggers (Onset Computer Corporation). We placed a single temperature logger within the thermal refuge where fish are known to aggregate and another logger approximately 100 m upstream of each refuge in the main stem. We fixed each temperature logger to a 6″ PVC pipe and railroad tie plate and placed each firmly into the river substrate. Temperature loggers recorded water temperature (°C) every 30 min throughout our study.

### 2.3 Camera trap image processing

**FIGURE 2:**
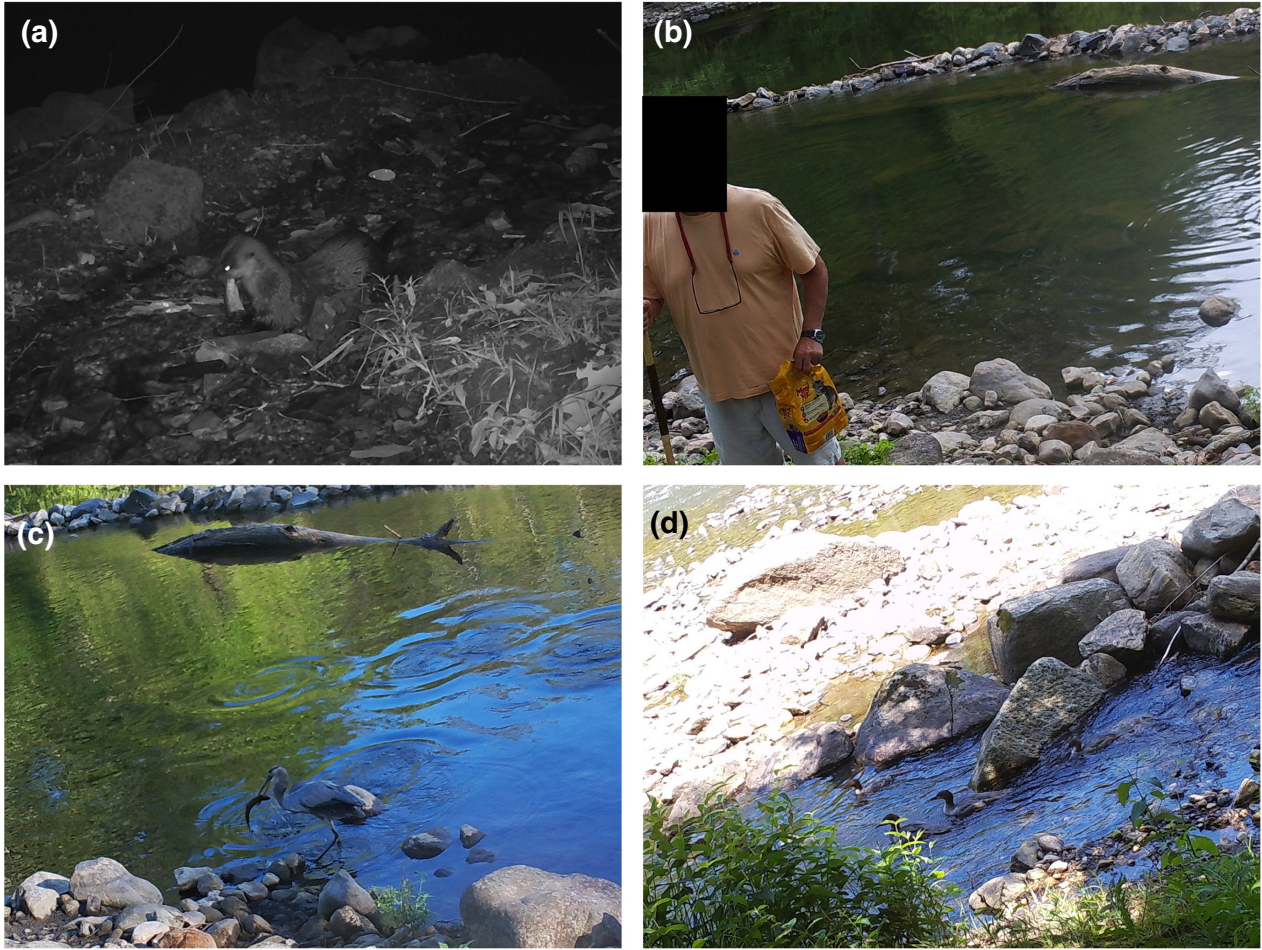
Examples of observations of different categories of terrestrial predators at thermal refuges along the Housatonic River, Connecticut, USA, including a river otter eating a brown trout (a), a non‐angler feeding brown and rainbow trout with commercial cat feed (b), a great blue heron with a brown trout in its' mouth (c), and several common mergansers swimming through a refuge (d).

We downloaded camera trap images and extracted terrestrial predator counts similar to other studies (e.g., Burton et al., 2022; O'Connor et al., 2017). We defined an independent observation as a three‐photo trigger event containing evidence of terrestrial predator presence in a single photo. We mandated that independent observations be separated by at least 30 min to be considered a unique observation on each camera trap and applied this restriction to all cameras deployed at a site. For example, if we observed an angler 20 min apart at two separate camera traps within a single site, this would be considered a single observation for that thermal refuge. We identified and enumerated observed terrestrial predator types (mammal, avian predator, and people) from each trigger‐event picture, recorded the date and the time of day, recorded the activity that people were engaging in (e.g., angling, swimming, launching a kayak, etc.), and noted (1 = yes, 0 = no) whether a predation attempt on a thermal refuge‐dwelling salmonid was occurring. In a concurrent study, >99.9% of all identified fish from the four thermal refuges were adult brown or rainbow trout (C.J. Sullivan, *unpublished data*). We, thus, defined a predation attempt as observing a predator remove an adult brown or rainbow trout from the thermal refuge. We separated all people into one of two groups, “angler” or “non‐angler”, where we designated an angler as a person wearing fishing gear (e.g., waders) and carrying a fishing rod and a non‐angler as someone without fishing gear. Figure 2 provides examples of identified terrestrial predators.

### 2.4 Statistical analysis

Preliminary data exploration revealed that we captured >10 species of terrestrial predators, in addition to anglers and non‐anglers, and some species were observed in only a few instances. Prior to analyses, we grouped some infrequently observed terrestrial predators into several major thematic groups, allowing for statistical inference for certain species. We aggregated coyote *Canis latrans*, red fox *Vulpes vulpes*, and bobcat *Lynx rufus* into a single “carnivore” category, and turkey vulture *Cathartes aura*, bald eagle *Haliaeetus leucocephalus*, and osprey *Pandion haliaetus* into a single “raptor” category. All other more frequently observed species were not aggregated into larger groups. We then omitted terrestrial predator categories with <5 individual observations.

To address our first objective, we first described diel terrestrial predator visitation rates averaged across all thermal refuges throughout the study period. We defined visitation rate as the average count of a terrestrial predator category across all thermal refuges during each hour of the day across the sampling period (count/h). We used a Rayleigh's test to determine whether terrestrial predator visitation patterns were uniformly distributed throughout a 24 h period by predatory category.

We used generalized additive mixed models (GAMMs) to evaluate the effects of terrestrial predator category and prevailing environmental conditions on mean daily hourly visitation rates (mean count/h/day). We used GAMMs because they simultaneously evaluate linear and nonlinear relationships between dependent and independent covariates, better representing ecological relationships rather than assuming linearity. We created a suite of environmental covariates representing prevailing conditions in the Housatonic River that we hypothesize could influence visitation patterns by different terrestrial predator types. We calculated the mean daily main stem Housatonic River water temperature (*T*_Hous_; °C) and the mean daily water temperature differential (*T*_diff_; °C) between the main stem river and each thermal refuge using data collected from HOBO temperature loggers. We obtained Housatonic River discharge data from a U.S. Geological Survey (https://usgs.gov/) gauging station at Falls Village (gaging station #01199000) restricted to the duration of our study and calculated the mean daily Housatonic River discharge (*D*_Hous_; m^3^/s). We also obtained daily maximum air temperatures (*T*_max_; °C), daily mean (*WS*_mean_) and maximum 5 s (*WS*_max_) wind speeds (kph), and daily total precipitation (*Precip*; mm) from National Oceanic and Atmospheric Administration (https://www.ncdc.noaa.gov/) gauging station in Pittsfield, Massachusetts (station #00014763). Each covariate is directly relevant to our study hypotheses and represents a different environmental condition that may be conducive for terrestrial predator activity and foraging in riverscapes.

We built GAMMs identifying potential factors influencing terrestrial predator visitation rates at thermal refuges using the “mgcv” tools package in Program R (version 4.2.2; R Development Core Team, 2023). We constructed models using all possible additive combinations of the seven environmental covariates as fixed effects with (thin plate regression spline; Wood, 2006) and without a smoothing function, a fixed effect for terrestrial predator category, and a random effect for Julian date. We included Julian date as a random effect because we were not interested in determining causal inference imposed by date but still needed to control its effects. Further, we included a binary variable for salmonid presence within thermal refuges gathered from underwater time‐lapse cameras at Furnace and Mill brook (July 1st to September 6th; C.J. Sullivan, *unpublished data*). We also constructed an additional set of models where we included interactions between each of the seven environmental covariates and the terrestrial predator category using all possible model combinations. We checked for correlation among environmental covariates using Pearson's correlation coefficient prior to the analysis; we excluded *T*_Hous_ and WS_max_ from the analysis due to high (>0.7) correlation coefficients with other covariates. We used maximum likelihood (ML) parameter estimates, compared to the default restricted ML (REML) estimates since REML estimates are not comparable across models when fixed effects differ (Zuur et al., 2009). We calculated the size‐corrected Akaike's information criterion (AIC_c_) and delta AIC_c_ (Δ_*i*_) to rank candidate models based on their support for the data. To control for autocorrelation, we applied an exponential correlation structure after comparing models with the following correlation structures using AIC_c_: exponential, Gaussian, linear, rational quadratics, and spherical (Pinheiro & Bates, 2000). We only interpreted models with ΔAIC_c_ < 2.0 and AIC_c_ weights >0.10 (Burnham & Anderson, 2003).

Last, we built a single GAMM model predicting the probability of a predation attempt on salmonids within thermal refuges by hour of the day. We constructed the model using a binary response variable indicating whether the observed terrestrial predator was predating an adult brown or rainbow trout (yes = 1, no = 0), and a single covariate, hour of the day (24 h). Due to the limited observations of predation attempts by all categories of terrestrial predators, we did not include a fixed effect for terrestrial predator category. We used the default smoothing function for the single covariate and applied the “binomial” family option to estimate a logistic‐style GAMM model. We used the default REML parameter estimates and applied an exponential correlation structure. Last, we converted predicted values to the probability scale using the “plogis” function and thus report the probability of predation by any category of terrestrial predator by hour of the day across all thermal refuges and sampling dates.

## 3 RESULTS

We collected a total of 2540 independent terrestrial predator photo events from camera traps at thermal refuges throughout the Housatonic River. We continuously monitored terrestrial predators at thermal refuges with no survey gaps because we had no camera trap malfunctions or dead batteries. The number of individual observations by terrestrial predator type is as followed: angler (*n* = 321), carnivore (*n* = 27), common merganser *Mergus merganser* (*n* = 189), great blue heron *Andrea herodias* (*n* = 678), non‐angler (*n* = 437), raccoon (*n* = 848), raptor (*n* = 31), and river otter (*n* = 9; Table 1). During monitoring, mean daily Housatonic River water temperature (*T*_Hous_) ranged from 17.95 to 27.29°C (mean ± 1 SE = 22.72 ± 0.07) and mean daily discharge (*D*_Hous_) ranged from 2.60 to 45.89 m^3^/s (11.77 ± 0.27). The mean daily water temperature differential (*T*_diff_) between the Housatonic River and monitored thermal refuges ranged from 0.61 to 6.12°C (3.55 ± 0.04). Daily maximum air temperatures (*T*_max_) ranged from 12.80 to 33.30°C (24.98 ± 0.13) and daily total precipitation (*Precip*) ranged from 0.00 to 33.53 mm (2.54 ± 0.18). Last, daily mean (*WS*_mean_) and maximum 5 s (*WS*_max_) wind speeds ranged from 0.00 to 19.79 (8.35 ± 0.13) and 17.70 to 72.40 kph (35.22 ± 0.33), respectively.

**TABLE 1:** Raw number of total observations, predation attempts, and mean (and SE) count per hour (count/h) for each category of terrestrial predator across the four thermal refuge sites located in the Housatonic River, Connecticut, USA monitored using camera traps between May 18th and September 29th, 2022.

Terrestrial predator category	No. observations	No. of predation attempts	Count/h	
			Mean	SE
Angler	321	3	0.0244	0.0014
Carnivore	27	0	0.0019	0.0004
Common Merganser	189	0	0.0502	0.0053
Great Blue Heron	678	10	0.0464	0.0018
Non‐Angler	437	0	0.0422	0.0022
Raccoon	848	8	0.0636	0.0024
Raptor	31	5	0.0030	0.0006
River Otter	9	3	0.0010	0.0004

**FIGURE 3:**
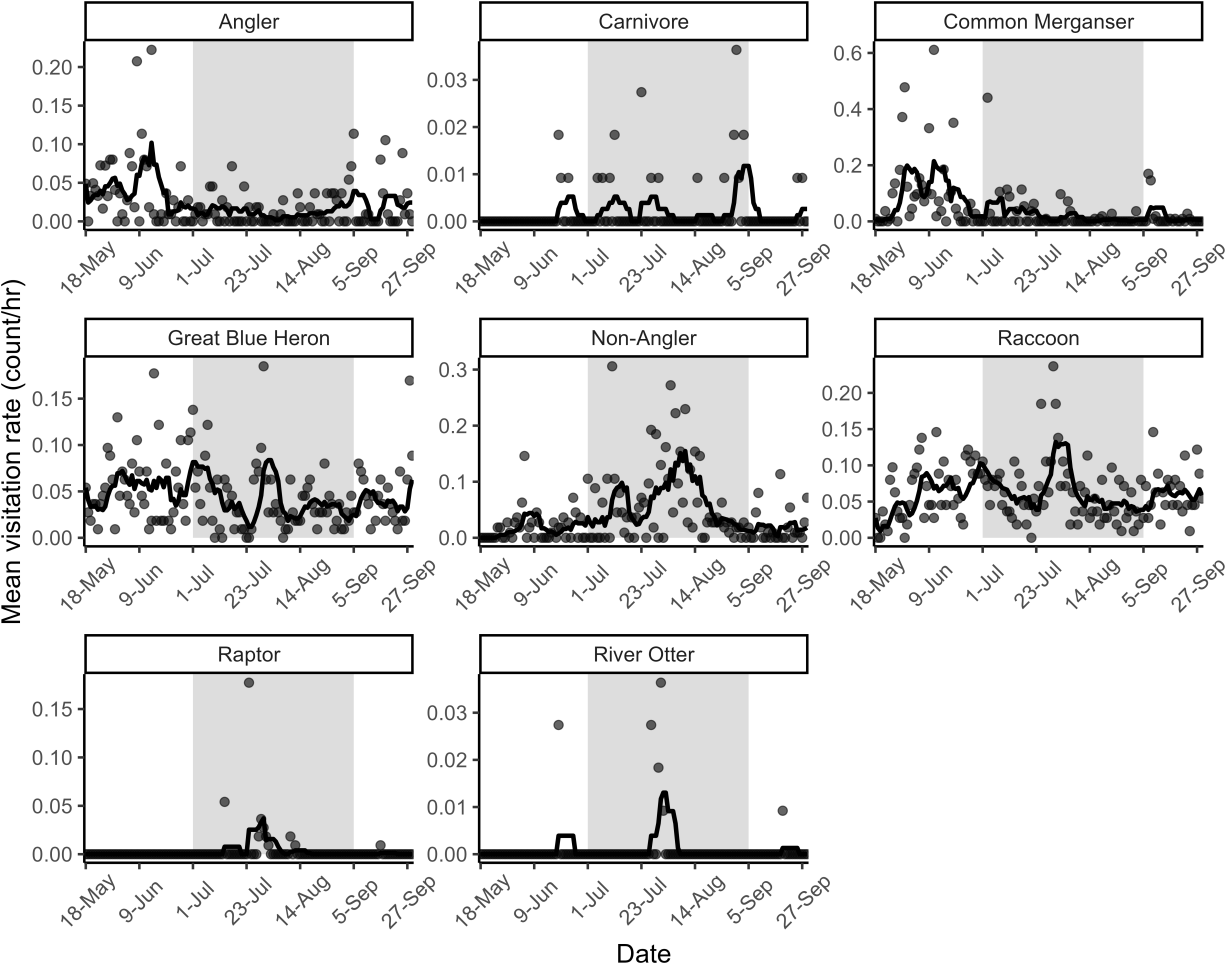
Mean hourly visitation rates (count/h/day) from May 18th to September 29th, 2022, of different categories of terrestrial predators captured from camera traps at thermal refuges within the Housatonic River, Connecticut, USA. The black line represents a moving average trend line. The gray‐shaded region represents the period when cold‐water‐dependent salmonids inhabited thermal refuges during the summer 2022 at Furnace and Mill brooks (C.J. Sullivan, *unpublished data*).

**FIGURE 4:**
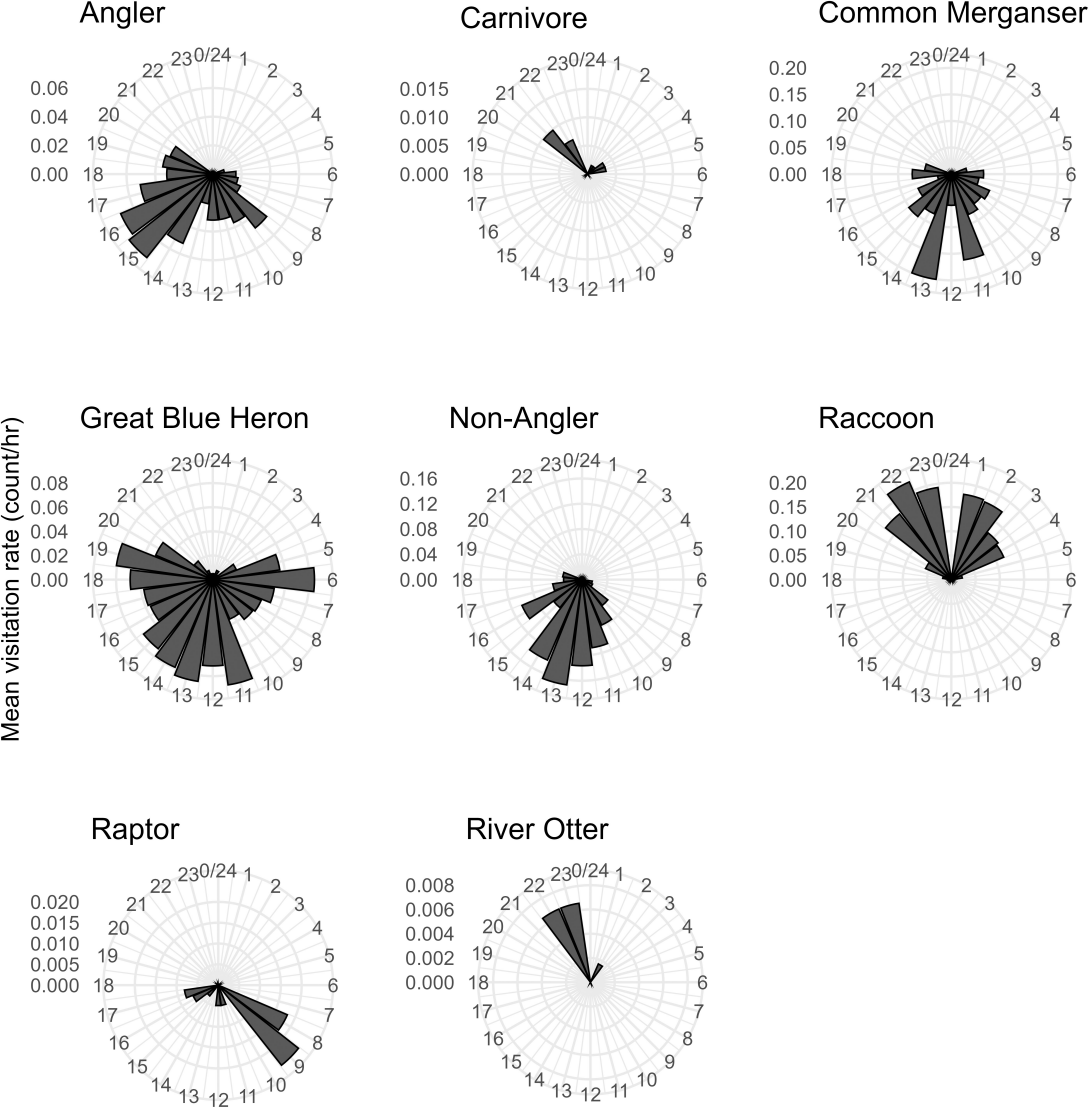
Hourly visitation rates (count/h) of different categories of terrestrial predators captured from camera traps at thermal refuges within the Housatonic River, Connecticut, USA, from May 18th to September 29th, 2022.

Mean hourly visitation rates (count/h) were variable across terrestrial predator categories and sampling dates (Figure 3). We detected at least one category of terrestrial predator at a thermal refuge each day of sampling and enumerated an average of 1.03 (SE = 0.008) predators during each observation. Across all categories of terrestrial predators, mean visitation rates (count/h) varied from 0.00 to 0.61 count/h throughout a 24 h period (Figure 4). Rayleigh's tests indicated that visitation rates were not uniform throughout a 24 h period for each category of terrestrial predator (all *p* ≥ .455), suggesting that predator visitations tended to be concentrated in time. Generally, mean visitation rates were highest between 12:00 and 17:00 for anglers and non‐anglers. Mean visitation rates for raccoons, carnivores, and river otters peaked between 21:00 and 22:00, and visitation rates peaked at 13:00 and 09:00 for common merganser and raptors, respectively. Last, mean visitation rates were consistently higher during daylight hours compared to nighttime hours for great blue heron.

**FIGURE 5:**
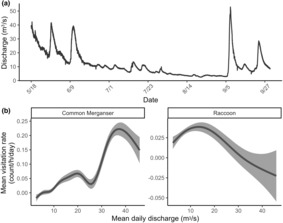
(a) Time series of the Housatonic River discharge (m^3^/s) from the U.S. Geological Survey gauging station at Falls Village (gaging station #01199000) between May 18th and September 29th, 2022. (b) Relationship between hourly visitation rates (count/h/day) of common mergansers (left) and raccoons (right) at thermal refuges and the mean daily Housatonic River discharge (*D*_Hous_; m^3^/s); *D*_Hous_ did not significantly influence the visitation rates of other categories of terrestrial predators. Gray polygons represent 95% confidence intervals (CIs).

**FIGURE 6:**
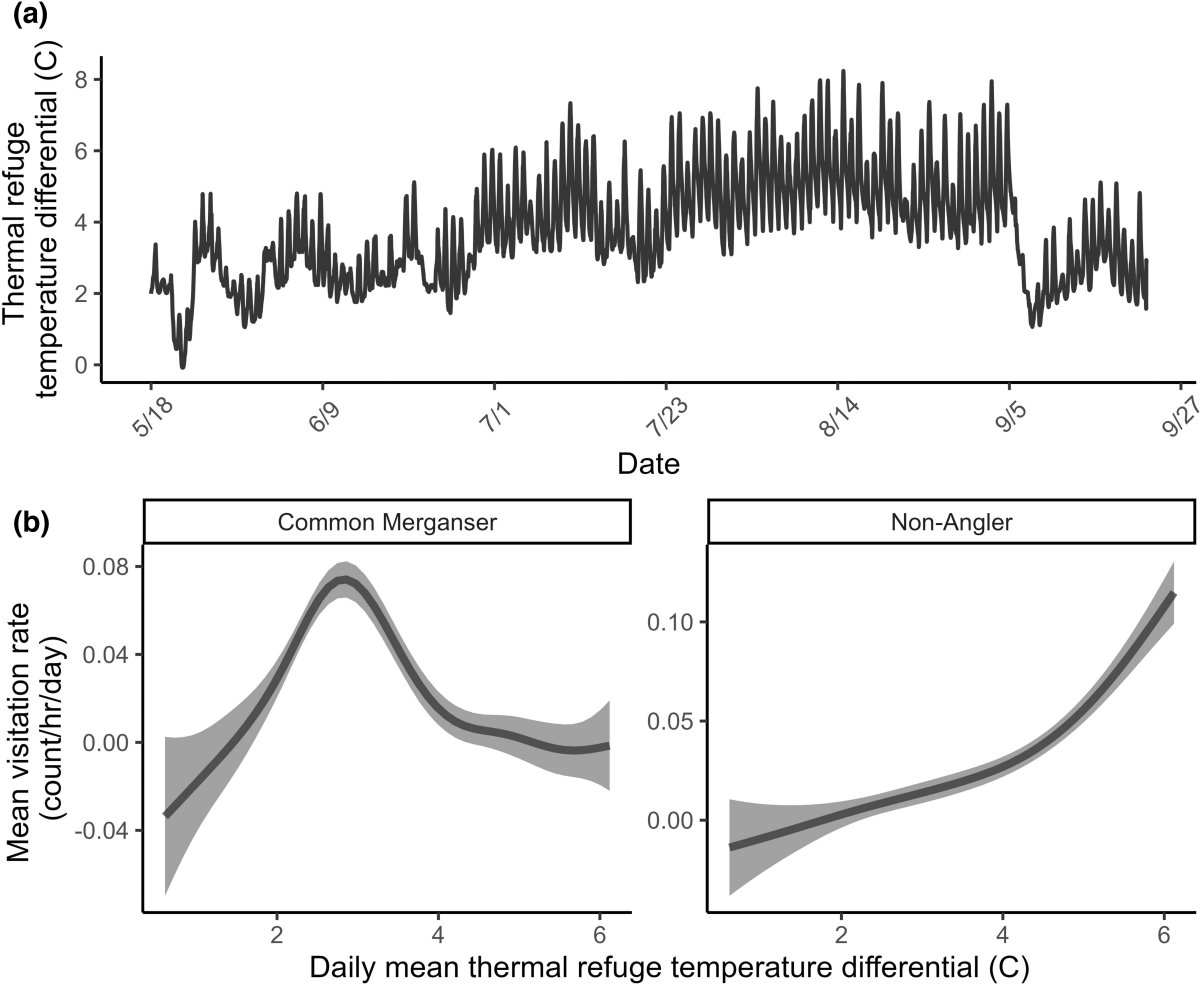
(a) Time series of the water temperature differential (*T*_diff_; °C) between the mainstem Housatonic River and thermal refuges between May 18th and September 29th, 2022, averaged across refuges. (b) Relationship between hourly visitation rates (count/h/day) of both common mergansers (left) and non‐anglers (right) at thermal refuges and the mean daily water temperature differential (*T*_diff_; °C); *T*_diff_ did not significantly influence the visitation rates of other categories of terrestrial predators. Gray polygons represent 95% confidence intervals (CIs).

The most supported model (AIC_c_ = −3764.72, AIC_c_ weight = 0.74; Table 2) indicated that mean terrestrial predator visitation rates (count/h/day) were best explained by variations in mean daily Housatonic River discharge (*D*_Hous_) and mean daily water temperature differential (*T*_diff_), and relationships varied by the category of terrestrial predator and were not significant for some (Table 3). Two categories of terrestrial predators exhibited significant and differing relationships with mean daily Housatonic River discharge (*D*_Hous_). Common merganser mean visitation rates were positively and nonlinearly associated with mean daily Housatonic River discharge (*D*_Hous_; edf = 7.50; *p* = <.001), and peaked at a discharge of approximately 37.00 m^3^/s (Figure 5). In contrast, raccoon visitation rates were negatively and non‐linearly associated with mean daily Housatonic River discharge (*D*_Hous_; edf = 3.02; *p* = .001) and peaked at a discharge of approximately 13.00 m^3^/s (Figure 5). Common merganser and non‐anglers were the only categories of terrestrial predators with significant relationships with mean daily water temperature differential (*T*_diff_). Mean visitation rates by both common merganser (edf = 7.91; *p* < .001) and non‐anglers (edf = 3.63; *p* < .001) were positively and nonlinearly associated with the mean daily water temperature differential (*T*_diff_) between the Housatonic River and thermal refuges, but merganser visitation rates peaked at a temperature differential of approximately 3.00°C (Figure 6). Relationships between other categories of terrestrial predator‐specific visitation rates (count/h/day) and *D*_Hous_ or *T*_diff_ were not significant (Table 3). All other model combinations received less support (ΔAIC_c_ > 2.0 or AIC_c_ weight >0.10; Table 2).

**TABLE 2:** Top five GAMMs identifying potential factors influencing terrestrial predator visitation rates at thermal refuges in the Housatonic River, Connecticut, USA between May 18th and September 29th, 2022.

Model structure	Df	AIC_c_	ΔAIC_c_	AIC_c_ weight
~ Predator Category + S(*D*_Hous_, by = Predator Category) + S(*T*_diff_, by = Predator Category)	43	−3764.72	0	0.74
~ Predator Category + S(*T*_max_, by = Predator Category) + S(*Precip*, by = Predator Category)	44	−3762.63	2.09	0.26
~ Predator Category + S(*D*_Hous_, by = Predator Category) + S(*T*_diff_, by = Predator Category) + S(*Precip*, by = Predator Category)	59	−3740.98	23.74	<0.01
~ Predator Category + Salmonid Presence + S(*D*_Hous_, by = Predator Category) + S(*T*_diff_, by = Predator Category) + S(*Precip*, by = Predator Category)	60	−3738.97	25.75	<0.01
~ Predator Category + S(*D*_Hous_, by = Predator Category) + S(*T*_diff_, by = Predator Category) + S(*T*_max_, by = Predator Category)	59	−3717.45	47.26	<0.01

*Note*: We include model fixed effects with or without a smoothing function (S), Akaike's information criterion corrected for small sample size (AIC_c_), the difference between the AIC_c_ value of the most supported model and model *i* (ΔAIC_c_), and the relative support for model *i* (AIC_c_ weight). We only interpreted models with ΔAIC_c_ <2.0 and AIC_c_ weights >0.10 (Burnham & Anderson, 2003).

**TABLE 3:** Fixed effects from the most supported general additive mixed model (GAMM; Table 2) predicting the mean daily hourly visitation rates (mean count/h/day) of different categories of terrestrial predators at thermal refuges within the Housatonic River, Connecticut, USA between May 18th and September 29th, 2022.

Parameter	Estimate	SE	*t*‐value	*p*‐value
Terrestrial predator				
Angler	0.023	0.003	6.866	<.001
Carnivore	−0.022	0.005	−4.545	<.001
Common Merganser	0.021	0.005	4.427	<.001
Great Blue Heron	0.022	0.005	4.618	<.001
Non‐Angler	0.017	0.005	3.451	<.001
Raccoon	0.039	0.005	8.122	<.001
Raptor	−0.021	0.005	−4.301	<.001
River Otter	−0.023	0.005	−4.686	<.001
Smoothing function of *D*_Hous_				
Angler	0.007	0.004	1.621	.105
Carnivore	−0.001	0.004	−0.257	.797
Common Merganser	−0.027	0.046	−0.589	.556
Great Blue Heron	0.005	0.004	1.148	.251
Non‐Angler	−0.005	0.004	−1.072	.284
Raccoon	0.0016	0.017	0.980	.327
Raptor	0.0003	0.004	0.081	.935
River Otter	0.0004	0.004	−0.083	.934
Smoothing function of *T*_diff_				
Angler	−0.004	0.004	−1.026	.305
Carnivore	0.0001	0.004	−0.033	.974
Common Merganser	−0.145	0.060	−2.408	.016
Great Blue Heron	0.0005	0.004	−0.125	.900
Non‐Angler	0.060	0.022	2.649	.008
Raccoon	0.007	0.005	1.456	.146
Raptor	0.003	0.004	0.700	.484
River Otter	0.0001	0.004	0.029	.977

**FIGURE 7:**
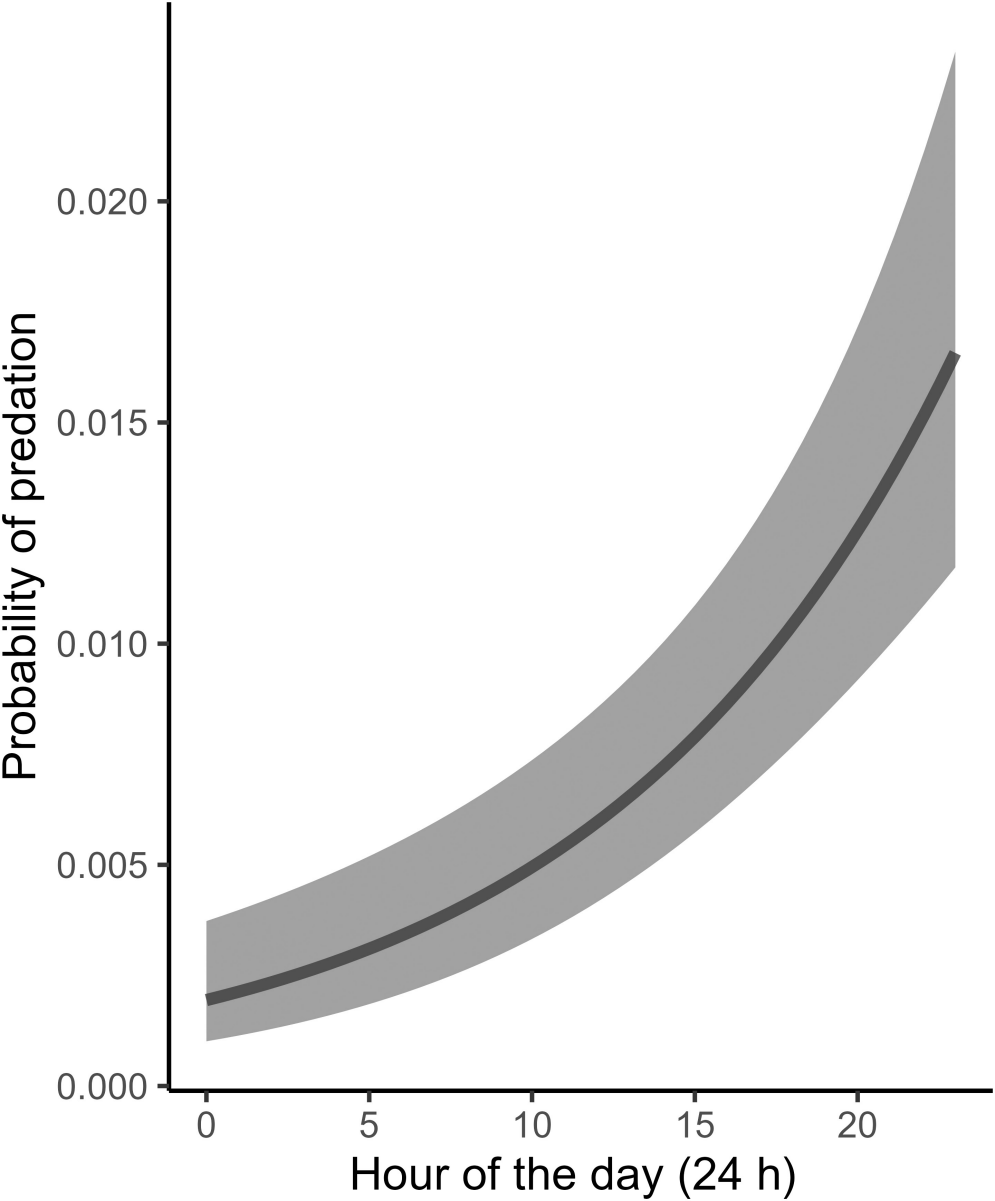
Prediction of the estimated probability of a predation attempt by any category of terrestrial predator, and 95% CIs, on aggregating cold‐water‐dependent salmonids across all thermal refuges within the Housatonic River, Connecticut, USA between May 18th and September 29th, 2022.

We observed a total of 22 separate predation attempts on brown and rainbow trout occupying thermal refuges (e.g., Figure 2 panels a, c). Predation attempts were most numerous by great blue herons (*n* = 9 predation attempts; 1.33% of total observations), followed by raccoons (*n* = 3; 0.35%), raptors (*n* = 5; 16.10%), and both anglers (*n* = 3; 0.93%) and river otters (*n* = 2; 22.22%; Table 1). Our GAMM model predicted that the probability of a predation attempt by any terrestrial predator increased from 0.002 to 0.017 throughout a 24 h day (*p* = .004) across all thermal refuges (Figure 7).

## 4 DISCUSSION

Camera traps provide novel evidence that terrestrial predators are pervasive at riverine thermal refuges, which is relevant for cold‐water fish and refuge conservation and management globally. Limited observations from previous studies investigating salmonid use of thermal refuges suggest that angling (Keefer et al., 2009) and avian (Ritter et al., 2020) predation pressures can be notably high at select refuges and times. We found that terrestrial predators were present at four thermal refuges along the Housatonic River at nearly all hours, and predation probabilities by all predator types were highest late in the day. Thermal refuges vary in their ability to provide effective relief from heat stress depending upon their configuration, temporal persistence, and distribution throughout a riverscape (Brewitt et al., 2017; Dugdale et al., 2013; Ebersole et al., 2015; Wilbur et al., 2020), and inter‐specific competition for food or locations within or near thermal refuges (Hitt et al., 2017; Morgan & O'Sullivan, 2023). We posit that terrestrial predator dynamics within and around thermal refuges may too drive refuge effectiveness given their chronic prevalence and, thus, represent a proximate threat to aggregating fish, but the indirect effects of predator presence may be greater than direct effects (i.e., predation mortality).

We observed a temporal partitioning of visits to thermal refuges by a suite of terrestrial predators, providing insights into the fine‐scale variation in perceived predation risks at refuges. Numerous species of terrestrial predators forage in riverscapes, including raptors (e.g., red‐tailed hawk *Buteo jamaicensis*; Harvey & Nakamoto, 2013), double‐crested cormorant *Phalacrocorax auratus* (Hostetter et al., 2012), American white pelican *Pelicanus erythrorhynchos* (Budy et al., 2022), river otter and American mink *Neovison vison* (Heggenes & Borgstrom, 1988), large carnivores (e.g., black bear *Ursus arctos* or coyote; Levi et al., 2015), and raccoon (Harvey & Nakamoto, 2013). We found a preponderance of terrestrial predator visitations at riverscape thermal refuges during the daylight or near‐dusk hours (Figure 4), corresponding well with previous literature. Harvey and Nakamoto (2013) and Metcalfe et al. (1999) found that >75% of terrestrial predator (not including humans) encounters with salmonids throughout riverscapes occurred during daylight hours. Riverscape thermal refuges are generally shallow, near‐shore habitats with little canopy and in‐stream cover (Ebersole et al., 2003) and their temperature differentials (and salmonid densities) are generally highest near dusk (Ebersole et al., 2001). It is, thus, logical to expect a diel visitation pattern by different terrestrial predator types at thermal refuges. For example, our observations suggest that angler, non‐angler, common merganser, great blue heron, and raptor (visual predators) can commonly visit thermal refuges during daylight hours whereas raccoon and river otter can be common nocturnal visitors (opportunistic predators). A key question moving forward is whether the diel, but nonetheless chronic, perceived risks imposed by terrestrial predators at thermal refuges differ from other river reaches or is amplified at select refuges or refuge types due to differential predator densities throughout the riverscape. Additionally, we did not individually identify and track individual terrestrial predators, and whether “problem” individuals may be disproportionately visiting refugees is an open question.

Daily Housatonic River conditions influenced visitation rates by some categories of terrestrial predators at the four thermal refuges, similar to other studies evaluating salmonid vulnerability to predation in riverscapes (e.g., Harvey & Nakamoto, 2013; Hostetter et al., 2012; Teuscher et al., 2015; Vehanen et al., 2021). We found that mean daily Housatonic River discharge (m^3^/s) and thermal refuge temperature differential (°C) influenced common merganser, raccoon, and non‐angler visitation rates, but environmental conditions were not influential otherwise (Figures 5 and 6). Low river discharge can increase foraging efficiency of riverine fish by terrestrial predators as river depth declines and fish movements become more limited (e.g., Hostetter et al., 2012), which can benefit predators that may forage opportunistically (e.g., raccoon). In contrast, we found that common merganser visitation rates were positively associated with mean daily Housatonic River discharge (m^3^/s), which could reflect a pursuit‐diving foraging strategy that is facilitated by higher discharge. Further, more salmonids will use thermal refuges as main stem water temperatures increase (Ebersole et al., 2001), increasing the size of fish aggregations in refuges. Non‐angler visitation rates increased with increasing main stem temperature differential (°C) as non‐anglers may be interested in viewing the unique phenomena of fish behavioral thermoregulation or swimming, and common merganser visitation rates peaked at a temperature differential of 3°C. Interestingly, the fixed effect for salmonid presence within thermal refuges was not included in the most supported GAMM, suggesting that terrestrial predators may regularly visit refuges or the larger area that refuges are nested within for other reasons. We conducted our study in a single, regulated, fifth‐order river in a roughly 15.0 km segment, and we anticipate that river conditions (and salmonid presence) may have greater influence in more free‐flowing rivers or across lower‐order rivers where conditions may vary more widely.

We detected few predation attempts (*n* = 22) by terrestrial predators on salmonids aggregating in thermal refuges, resulting in a generally low predation probability. Terrestrial predators, both human and animal, commonly predate on trout and salmonids and can be notably high in shallow locations within riverscapes (e.g., Budy et al., 2022; Harvey & Nakamoto, 2013). Since salmonid density in thermal refuges can increase during the hotter late afternoon (Ebersole et al., 2001), it is no surprise that the probability of predation increased throughout the day. We did not separate predation probabilities by terrestrial predator type, but the low occurrence of predation attempts may reflect the general ability of salmonids to avoid predators or escape capture in thermal refuges. Salmonids are fast swimming, have high initial burst swimming speeds (Farrell, 2007), and can begin to flee terrestrial predators at distances >10 m (C.J. Sullivan, *personal observation*). We anecdotally noticed salmonids would temporarily leave thermal refuges as researchers approached, would remain skittish upon their return, and sometimes inhabit deeper (>1.0 m) portions of the refuge if available. Salmonid behavioral responses to terrestrial predator threats above the water's surface, thus, likely reduce their vulnerability to predation but could result in individual fish expending more energy if forced to temporarily occupy the hotter main stem river (e.g., Preisser et al., 2005; Ritter et al., 2020). There may too be variations in individual salmonid vulnerabilities to terrestrial predators based on body mass or boldness (e.g., Härkönen et al., 2014; Tsuboi et al., 2016). For example, Ryan et al. (2003) found that avian predators predated upon larger steelhead at higher rates compared to smaller steelhead, potentially due to their size and affinity for orienting near the water's surface (Collis et al., 2001). It may also be that more recently stocked salmonids are more vulnerable than wild or even “holdover” salmonids due to a lack of innate or learned predator avoidance behaviors (Olla & Davis, 1989), more frequent orientation near the water's surface (Mason et al., 1967), or increased stress levels if recently stocked (Sigismondi & Weber, 1988).

Selective predation (i.e., the targeting of individual fish) by terrestrial predators can result from several conditions, including differences in predator–prey encounter rates and foraging approach or capture rates by predators (Temple, 1987). Terrestrial predators could also selectively predate upon fish in poorer condition to increase predation efficiency, expending less energy to capture less‐responsive fish than healthy fish (e.g., Hostetter et al., 2012). Aggregating salmonids distribute based on size and species (Morgan & O'Sullivan, 2023) and interspecific interactions (Hitt et al., 2017) in thermal refuges. Additionally, an individual salmonid's previous thermal history (e.g., O'Sullivan et al., 2023) or condition (e.g., wounding via a failed predation attempt; e.g., Hostetter et al., 2012) could influence its location within a thermal refuge. We did not address selective predation on salmonids by terrestrial predators within thermal refuges herein, but we frequently noted wounded salmonids in thermal refuges. Many of the observed predation attempts occurred within the shallow fringes of thermal refuges (see Figure 2 panel c), suggesting that terrestrial predators may be targeting the few fish in poorer conditions (e.g., wounded). We estimated that <200 salmonids occupied the four thermal refuges during late‐July to early‐August (C.J. Sullivan, *unpublished data*), and even a limited amount of predation attempts could affect a large proportion of the thermoregulating population. Selective predation could, thus, further mitigate the thermoregulatory benefits of thermal refuge use for more vulnerable individuals or species in marginal riverscapes, and given our observations, we recommend more rigorous investigations into such mechanisms.

Proactive and centralized thermal refuge conservation will be necessary for the coming decades as riverscapes become more marginalized for cold‐water‐dependent salmonids. Our results suggest that managing or mitigating terrestrial predator visitations to thermal refuges, and even the small amount of direct predation mortality, may improve refuge effectiveness for salmonids more readily. Current thermal refuge management actions primarily aim to enhance the spatial extent and preserve refuge water temperatures by erecting current‐deflecting rock walls or deepening areas where fish aggregate (Kurylyk et al., 2015), which could indirectly reduce predation risks by expanding aggregating areas and habitats. Managers could also plant trees along the shoreline to cool thermal refuge water temperatures (e.g., Ebersole et al., 2003) but may invite terrestrial predators to perch or hunt closer to refuges. Installing large cobbles and boulders within thermal refuges could shelter salmonids from terrestrial predators (Bradford & Higgins, 2001) and broken water surfaces from large boulders could further conceal fish, or provide predators with greater access within refuges. Other examples of more temporary actions could be installing an array of wires or fixed instream woody additions (e.g., large logs fixed to anchors; e.g., Kurylyk et al., 2015). Angler and non‐angler visitations accounted for 29.8% (758/2540) of total terrestrial predator visits to study thermal refuges, suggesting that management and conservation agencies consider management interventions to mitigate potential negative outcomes of human presence. The CT DEEP prohibits angling within 100 ft of select thermal refuges from June 1st to September 15th using signs posted to nearby trees, but illegal angling still occurs. We suggest that human activities must be further restricted (e.g., no swimming and farther set‐back distances). Allowing actual or perceived impacts from terrestrial predators (both human and animal) to go unmitigated could lead to escalating social or political pressures or declining confidence toward conservation objectives by interested parties. Thermal refuge conservation within state, provincial, and federal agencies should include actions to mitigate terrestrial predator effects on thermoregulating salmonids when appropriate, focusing on indirect effects, but must also consider that most are federally protected or not hunted/trapped.

Camera traps provided unique insight into the ecology of terrestrial predators visiting and foraging in riverscape thermal refuges. Research investigating terrestrial predator effects on riverine fish is generally labor‐ and cost‐intensive and often involves handling and processing (i.e., tagging or diet extraction) a subset of both predators and individual fish (e.g., Budy et al., 2022; Ritter et al., 2020). Such activities in thermal refuges could be particularly controversial as disturbing or capturing refuge‐dwelling fish can result in temporary displacement (Price & Peterson, 2010) or unintentional mortality (Snyder, 2003), challenging managers and researchers to develop and implement less‐stressful survey methods (e.g., Struthers et al., 2015). Alternatively, Keefer et al. (2009) used long‐term tag return data to conclude that adult summer steelhead that used thermal refuges were harvested at a higher rate than fish using the main stem river, but these data rely on commercial, sport, and tribal fish harvest which may not be permitted or encouraged elsewhere. Camera traps (<$500 USD) offer an opportunity to monitor all terrestrial predator activities cheaply and passively across many thermal refuges concurrently, negating the need to handle animals. Camera traps can also be deployed prior to the warm summer months, eliminating potential disturbances from researchers repeatedly visiting refuges. Similar to regional and global camera trap monitoring networks emerging across terrestrial landscapes, we recommend the implementation of a coordinated monitoring network across thermal refuges using camera traps. We primarily focused on when and how many terrestrial predators visited thermal refuges per hour of the day. The same surveys can also generate data on predator behaviors (e.g., actively feeding or not, directional movement or space use, or prey size or species selectivity), further enriching our understanding of cumulative predator effects in refuges across diverse and complex riverscapes.

Thermal refuges provide opportunities for cold‐water‐dependent salmonids to resist warming and heatwaves associated with climate change (Kurylyk et al., 2015; Morgan & O'Sullivan, 2023; Sullivan et al., 2021), and a single refuge can be important for the survival of many individuals throughout the larger riverscape (Barret & Armstrong, 2022). In contrast to predation mortality, high levels of terrestrial predator visitation rates can cause fish to avoid certain areas or change their activity schedules (e.g., Doherty et al., 2021; Rahel & Stein, 1988), and short‐term avoidance of thermal refuges by individual salmonids after repeated encounters with predators could deplete energy resources necessary for summer survival (e.g., Preisser et al., 2005). Our findings suggest that terrestrial predators are pervasive at thermal refuges, and repeated encounters over time may eventually lead to fish avoiding critical refuges at certain times of the day (e.g., later in the day when predation probabilities are highest). In such situations, conservation biologists must consider management interventions to alter or mitigate predator visitation (and predation rates) at critical riverine thermal refuges. Future research should investigate the predator–prey behavioral interactions of cold‐water‐dependent salmonids and terrestrial predators within thermal refuges and to what degree perceived predation risks might reduce individual fish fitness and survival if refuges are avoided intermittently or at certain times of the day. We would also encourage researchers to determine the degree to which terrestrial predators (mammal or avian) may depend on aggregating salmonids in thermal refuges for summertime forage, similar to other fish aggregation phenomenon (e.g., Pacific salmon *Oncorhynchus* spp. migrations).

## AUTHOR CONTRIBUTIONS

**Christopher J. Sullivan:** Conceptualization (lead); data curation (lead); formal analysis (lead); funding acquisition (lead); investigation (lead); methodology (lead); software (equal); validation (equal); visualization (lead); writing – original draft (lead); writing – review and editing (equal). **Chadwick D. Rittenhouse:** Conceptualization (supporting); formal analysis (supporting); methodology (supporting); writing – review and editing (supporting). **Jason C. Vokoun:** Conceptualization (supporting); data curation (supporting); formal analysis (supporting); funding acquisition (supporting); investigation (supporting); methodology (supporting); project administration (lead); resources (lead); software (supporting); supervision (lead); validation (equal); writing – review and editing (equal).

## CONFLICT OF INTEREST STATEMENT

The authors have no conflict of interest.

## FUNDING INFORMATION

This project was funded through the Storrs Agricultural Experiment Station, Hatch Act (grant/award number: CONS01039) and the State and Tribal Wildlife Grants program in cooperation with the Connecticut Department of Energy and Environmental Protection Fisheries Division (grant/award number: T21R1 SWG).

## Data Availability

Data that support the findings of this study are openly available at: https://github.com/csulliv51/Camera_Trap_Data_VisitationRates.
